# Invasive ductal carcinoma and small lymphocytic lymphoma/chronic lymphocytic leukemia manifesting as a collision breast tumor: A case report and literature review

**DOI:** 10.1515/biol-2021-0093

**Published:** 2021-08-27

**Authors:** Xiaowen Chen, Jianli Chen, Sihai Liao, Yuwen Cao

**Affiliations:** Department of Oncology Center, Affiliated Hospital of Guangdong Medical University, Zhanjiang, Guangdong, China; The Third Department of Medical Oncology, The Third Affiliated Hospital of Xinxiang Medical University, Xinxiang, Henan, China; Department of Pathology, Shihezi University School of Medicine, Shihezi, Xinjiang, China

**Keywords:** collision tumor, breast carcinoma, lymphoma, invasive ductal carcinoma, SLL/CLL

## Abstract

Collision breast tumors, consisting of breast cancer (BC) and non-Hodgkin’s lymphoma (NHL), are extremely rare. Here we report the case of a 64-year-old woman with a collision tumor in her left breast mass that was composed of invasive ductal carcinoma and small lymphocytic lymphoma/chronic lymphocytic leukemia. In addition, we reviewed the published comparable English-language literature. Collision breast tumor composed of BC and NHL is extremely rare. For that reason, there is a lack of consensus about the underlying mechanism, and diagnosing it without delay remains a complex clinical challenge. We found that post-menopausal, age-related estrogen levels changes and Epstein-Barr virus infection are possible pathogenic factors. However, the symptoms are almost identical, and it is difficult to distinguish a simple breast tumor from a breast collision tumor. In this study, we reviewed the clinical features of all patients with BC and NHL colliding breast tumors; this information might enable early identification and prevention of misdiagnosis.

## 1 Introduction

Synchronous breast cancer (BC) and non-Hodgkin’s lymphoma (NHL) is rare, and only 38 cases have been reported in the literature [1]. BC and NHL presenting in the same breast as a collision tumor is extremely rare. Collision tumor is the concrescence of two histologically distinct tumor subtypes occurring in the same site. Herein we report the case of a 64-year-old woman presenting with a collision tumor composed of invasive ductal carcinoma (IDC) and small lymphocytic lymphoma/chronic lymphocytic leukemia (SLL/CLL) in her left breast mass. To the best of our knowledge, only four such cases have been reported thus far [2–5].

## 2 Case report

**Figure 1:**
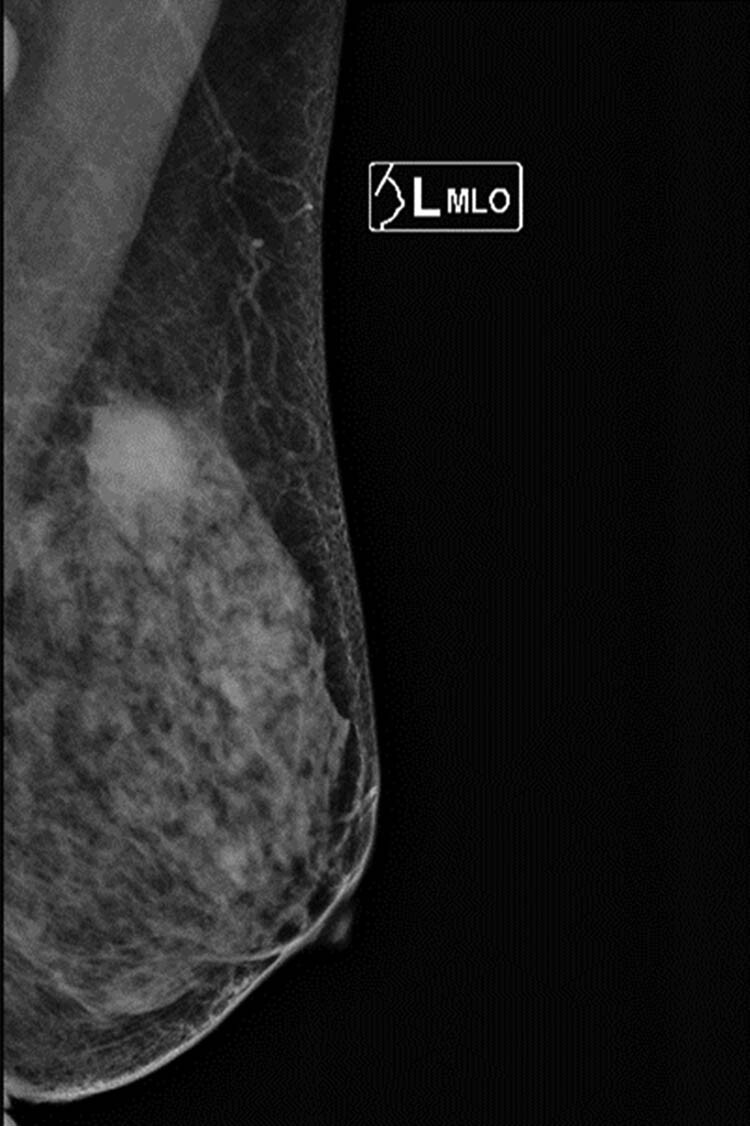
The mass is shown in mammary gland molybdenum target as a mass-shape high-density shadow.

**Figure 2:**
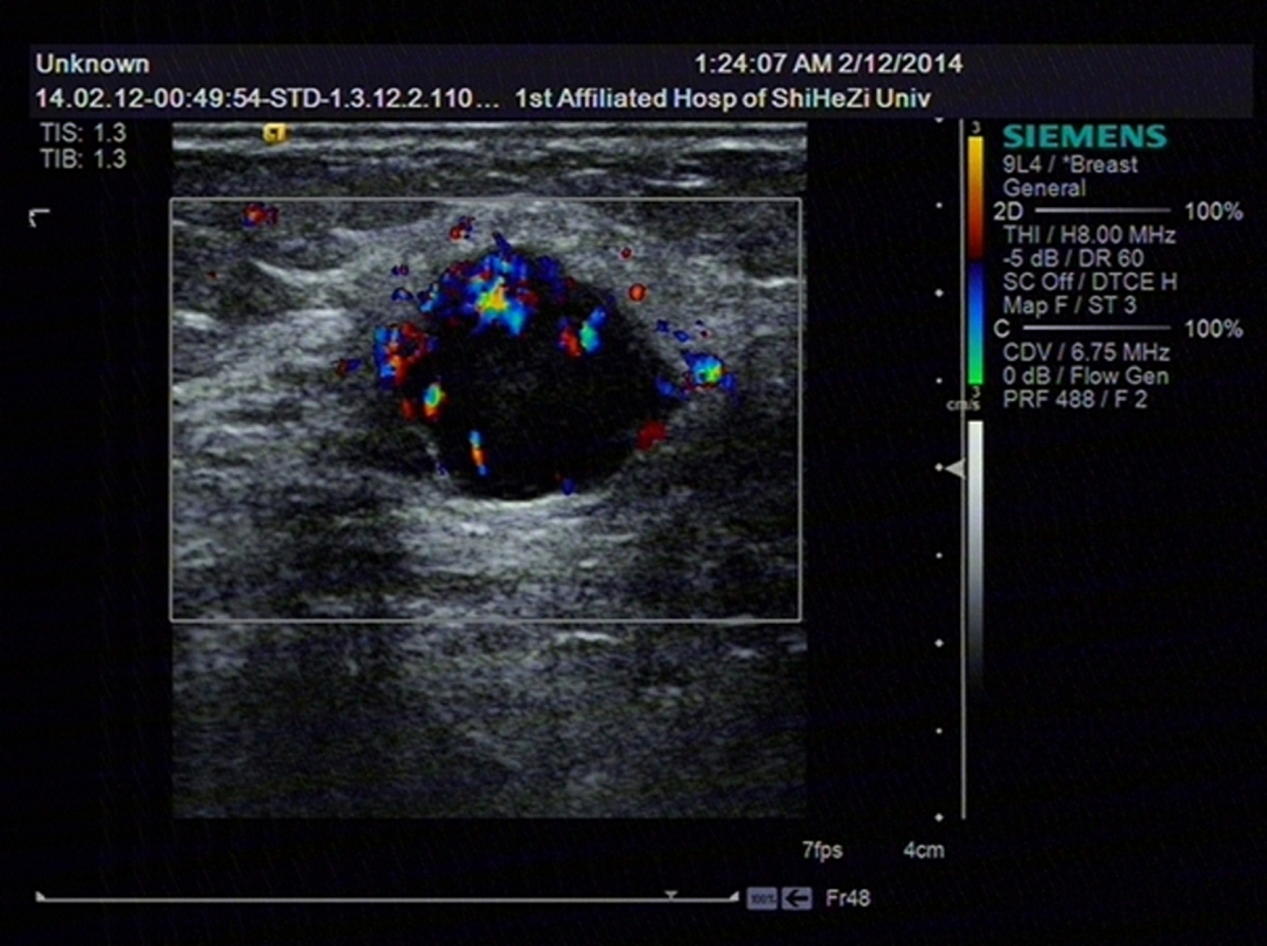
The left breast mass is shown in ultrasound as a low echo with a size of 2.4 cm × 1.5 cm.

A 64-year-old menopausal woman went to see a doctor to find a mass, which was nearly 2 cm in diameter and could be felt in the upper outer quadrant of the left breast. It is shown in mammary gland molybdenum target as a mass-shape high-density shadow (Figure 1) and shown in ultrasound as a low echo with a size of 2.4 cm × 1.5 cm (Figure 2) at 1–2 o’clock directions of the left breast. No abnormality was found in the right breast. There is a swollen lymph node at the left axillary, which is about 2.8 cm × 1.6 cm. Blood flow signals are visible in both masses. The fine needle aspiration (FNA) inspection on axillary lymph nodes (ALN) did not prompt malignant.

**Figure 3:**
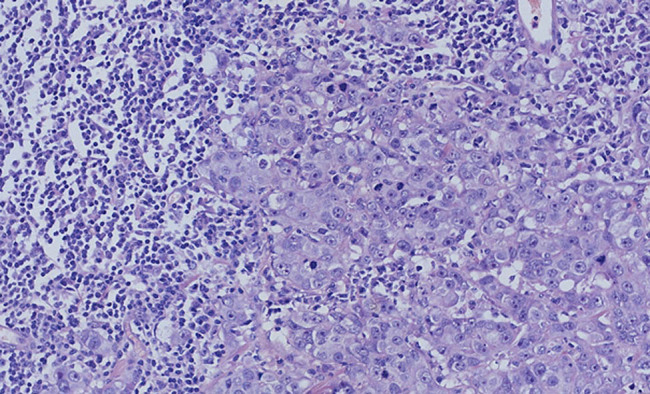
The left breast mass, IDC and diffuse proliferation of atypical lymphoid cells (HE, ×20).

**Figure 4:**
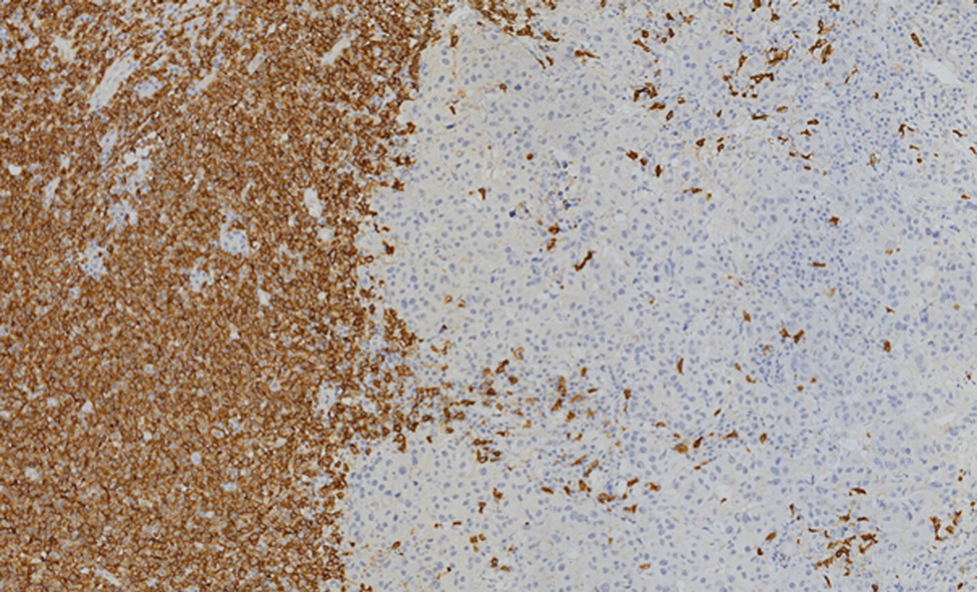
The left breast mass; lymphoid cells were CD20 positive (IHC, ×20).

The left breast mass histopathologic examination revealed collision tumors composed of IDC and SLL/CLL. IDC and diffuse proliferation of atypical lymphoid cells were visible (Figure 3), the morphology of lymphocyte with the characteristics of a single form, small to medium size, small round cells, less cytoplasm, smaller nuclear chromatin, less obvious nucleolus and visible mitotic count. Immunohistochemical staining shows IDC was ER, PR and Her-2 negative, while the atypical lymphoid cells were positive for CD20 (Figure 4) and CD23, but negative for CD3, CD5 and CyclinD1. Left ALNs and bone marrow were consistent with SLL/CLL, without BC metastasis. Microscopically atypical lymphoid cells with diffused hyperplasia was shown, and no cancer cell has been seen. The immunohistochemical staining is similar to that of atypical lymphoid cells in the left breast mass.

After a multidisciplinary discussion, we suggested chemotherapy, but she refused and took traditional Chinese medicine as a treatment instead. In 20 months, no evidence could indicate the disease progression.

**Informed consent:** Informed consent has been obtained from all individuals included in this study.**Ethical approval:** The research related to human use has been complied with all the relevant national regulations, institutional policies and in accordance with the tenets of the Helsinki Declaration, and has been approved by the authors’ institutional review board or equivalent committee.

## 3 Discussion

NHL as a second primary tumor occasionally occurs secondarily in BC patients receiving radiotherapy and chemotherapy. Synchronous BC and NHL are rare, and only 39 cases have been reported thus far in the English literature. BC and NHL are commonly found in different organs or lymph nodes and rarely occur in the same organ [1]. Collision tumor refers to the tumor formed by two primary tumors infiltrating each other, i.e., when two separate tumors occur in the same site. Carcinosarcoma is a rare form of collision mammary cancer with mixed epithelial and sarcomatoid components, accounting for <0.1% of all breast malignancies [6]. We report a case of colliding breast neoplasm consisting of BC and NHL that is much rarer than carcinosarcoma. To the best of our knowledge, only four such cases have been reported in the English literature [2–5]. Owing to the rarity of the disease, there is no consensus on its etiological mechanism and clinical characteristics, and early diagnosis remains a challenge for clinicians. We have presented a review of all these cases in Table 1.

**Table 1:** Collision breast tumors composed of BC and NHL

			BC					NHL		
Case	Gender	Age	Site	Type	Grade	ER/PR/Her-2	Metastasis	Site	Type	Metastasis
2004	Female	79	LB	IDC	NS	NS	LAN	LB	MALT	LAN and BM
2006	Female	53	LB	IDC	PD	NS	NS	LB	MALT	LSN
2007	Female	55	RB	IDC	WD	+/+/−	LSN	RB	SLL/CLL	RAN and BM
2015	Female	71	RB	IDC	WD	NS	NS	BB	SLL/CLL	RAN
Present case	Female	64	LB	IDC	PD	−/−/−	NS	LB	SLL/CLL	LAN and BM

BC, breast cancer; NHL, non-Hodgkin lymphoma; LB, left breast; BB, bilateral breast; RB, right breast; +, positive stated; −, negative stated; NS, not stated; PD, poorly differentiated; WD, well differentiated; RAN, right axillary node; LAN, left axillary node; BM, bone marrow; LSN, left sentinel node; MALT, mucosa-associated lymphoid tissue; SLL/CLL, small lymphocytic lymphoma/chronic lymphocytic leukemia.

The mechanisms that cause such collisions are very complex, and the pathophysiological association between the two concurrent tumors may be attributable to the fact that they are induced by the same causal factor. We noted that the average age of all 5 female patients was 64 years, and all of them were post-menopausal. NHL is more likely to occur in post-menopausal women [7], and estrogen increases the risk of BC [8,9]. Fats in the breast can increase estrogen biosynthesis, and the estrogen concentration in the breast remains relatively high despite the extremely low post-menopausal estrogen levels [10]. One possibility is that abnormally high estrogen levels in a post-menopausal woman’s breast can induce cancer and collision. It is important to explain that BC and NHL collision tumor occurs in the breast and not in other organs. Our study demonstrates the potential epidemiological factors that play a role, including age-related estrogen levels.

The other leading hypothesis is that certain viruses are simultaneously pathogenic in two different types of primary tumors, causing the collision. Long-term infection by the human papillomavirus causes colliding tumors in various organs, including the tongue [11] and the thyroid gland [12] as well as the vulva [13]. However, the Epstein-Barr virus (EBV) has been neglected for a long time. Our study suggests that EBV plays an important role in the collision between BC and NHL in the mammary glands. EBV infection promotes the occurrence of BC [14] and NHL [15] and particularly increases the risk of CLL occurrence [16]. It has been reported that there is a higher chance of detecting an EBV sequence in the IDC tissue than in a normal breast tissue [17]. The combination of IDC and SLL/CLL was more common in all the cases that we reviewed. This evidence suggests that EBV infection is an important inducer of BC and NHL colliding tumors.

The preoperative identification of the two tumor components in breast tumors is necessary because the treatment of BC and NHL is completely different. However, owing to similar clinical symptoms, colliding breast tumor is more likely to be mistaken for simple BC. In all the cases that we reviewed, it was often difficult to make a preoperative diagnosis using non-invasive imaging or with minimally invasive FNA. Yin et al. found that positron emission tomography (PET)/computed tomography may be more sensitive for identifying two different components in colliding tumors because of their uptake rate differences, demonstrating a mass with an increased uneven 18F-FDG uptake [18]. For collision breast tumors of BC and NHL, the use of some specially formulated contrast agents may be helpful for differentiating. The potential of 68Ga-NOTA-F (ab′)2-rituximab and 68Ga-NOTA-F (ab′)-rituximab as PET imaging agents for NHL has been reported [19].

Our case review showed that it is challenging to diagnose BC/NHL colliding breast tumors even with the post-operative pathology. Among the 100 collision tumors, the most common non-hematological neoplasms associated with a hematolymphoid proliferative disorder (HLPD) were from the breast (15%), and the most commonly identified HLPD was CLL/SLL (18%) [20]. In this cohort, 5% of the low-grade HLPDs, all of the CLL/SLL, were missed at initial sign-out. It is important to consider the collision of low-grade HLPDs before assuming that the lymphoid infiltrates represent an immunological response.

## 4 Conclusion

The combination of BC and NHL in collision breast tumors is rare. The mechanisms that cause collision breast tumors are very complex, and we do not yet completely understand the key causative factors. Clinical diagnosis of such cases is a serious challenge, and very few cases have been reported so far; therefore, we need to continue reporting such cases to share more useful information. Herein we reviewed the clinical features of all such cases of breast collision tumors for early identification and prevention of misdiagnosis.
